# High Gain Triple-Band Metamaterial-Based Antipodal Vivaldi MIMO Antenna for 5G Communications

**DOI:** 10.3390/mi12030250

**Published:** 2021-02-28

**Authors:** Tale Saeidi, Idris Ismail, Sima Noghanian, Adam R. H. Alhawari, Qammer H. Abbasi, Muhammad Ali Imran, M. Y. Zeain, Shahid M. Ali

**Affiliations:** 1 Electrical and Electronic Engineering Department, Universiti Teknologi PETRONAS, Bandar Seri Iskandar 32610, Malaysia; tale_g03470@utp.edu.my (T.S.); shahid_17006402@utp.edu.my (S.M.A.); 2 Wafer LLC, 2 Dunham Rd, Beverly, MA 01915, USA; 3 American Public University System, 111 W Congress St, Charles Town, WV 25414, USA; 4 Electrical Engineering Department, College of Engineering, Najran University, Najran 1988, Saudi Arabia; aralhawari@nu.edu.sa; 5 James Watt School of Engineering, University of Glasgow, Glasgow G128QQ, UK; Qammer.Abbasi@glasgow.ac.uk (Q.H.A.); muhammad.imran@glasgow.ac.uk.lectrical (M.A.I.); 6 Centre for Advanced Computing Technology (C-ACT), Faculty of Information and Communication Technology, Universiti Teknikal Malaysia Melaka, Hang Tuah Jaya, Durian Tunggal 76100, Malaysia; gammmd3@gmail.com

**Keywords:** MIMO antenna, 5G communication, X-band antennas, antipodal antennas, Vivaldi antennas

## Abstract

This paper presents a miniaturized dual-polarized Multiple Input Multiple Output (MIMO) antenna with high isolation. The antenna meets the constraints of sub-6 GHz 5G and the smartphones’ X-band communications. A vertically polarized modified antipodal Vivaldi antenna and a horizontally polarized spiral antenna are designed and integrated, and then their performance is investigated. Three frequency bands of 3.8 GHz, 5.2 GHz, and 8.0 GHz are considered, and the proposed dual-polarized antenna is studied. High isolation of greater than 20 dB is obtained after integration of metamaterial elements, and without applying any other decoupling methods. The proposed triple-band metamaterial-based antenna has 1.6 GHz bandwidth (BW) (2.9 GHz–4.5 GHz), 13.5 dBi gain, and 98% radiation efficiency at 3.8 GHz. At 5.2 GHz it provides 1.2 GHz BW, 9.5 dBi gain, and 96% radiation efficiency. At 8.0 GHz it has 1 GHz BW, 6.75 dBi gain, and 92% radiation efficiency. Four antenna elements (with eight ports) were laid out orthogonally at the four corners of a mobile printed circuit board (PCB) to be utilized as a MIMO antenna for 5G communications. The performance of the MIMO antenna is examined and reported.

## 1. Introduction

In cellular communication, when the number of users increases, because of insufficient channel bandwidth, the frequency provision becomes inadequate. The number of users cannot exceed a specific limit within the same frequency band. Moreover, with an increase in the number of users, co-channel interference increases. The capacity of sending and receiving data using 3G and 4G frequency channels is limited, making it difficult to stream videos and send large files. There is a need to have larger bandwidths and faster communication channels [1]. Two of the greatest advancements in increasing the bandwidth is the 5G communication and the use of multiple antennas in Multiple Input Multiple Output (MIMO). MIMO antennas can be utilized to increase the communication capacity through diversity and spatial multiplexing. Massive MIMO and beamforming of antenna patterns can offer better signal to noise ratio and reduce the power usage, especially at the time of utilizing a compact battery in small and handheld devices [2,3].

The standards for 5G are still under review, but there are several candidate bands. Some of the frequency bands that are considered for 5G communications are 3 GHz, sub- 6 GHz, and higher frequency bands at millimeter-wave (mm-Wave) bands [4,5]. To meet the high data rate requirements, the MIMO antenna system plays a critical role [6]. There has been an evolution in utilizing MIMO antennas in the current handsets to work at sub-3 GHz. However, this requires a larger area in handset devices. This problem can be solved by designing a multiband MIMO antenna so multiple working bands are covered by the integration of multiple antennas in one area [7,8]. However, the smaller the electrical size of the antenna is, the higher its Q factor, and, therefore, the narrower the antenna bandwidth is. This is a significant constraint for antenna design in a compact size device with a small form factor. Besides, there is a physical limit on the antenna radiation efficiency. The main challenge in the design of small antennas is to overcome the limited radiation efficiency.

Another challenge in MIMO system implementation is the mutual coupling effect between multiple antennas, an effect that is well-known in antenna array design. When multiple antennas are closely spaced (such as in compact devices), the electromagnetic waves radiated from one antenna interacts with its adjacent antennas. This mutual coupling effect will distort antenna radiation patterns, change the antenna’s impedance matching, and cause correlation in the transmitted or received signals, which directly corresponds to the reduction in MIMO system capacity [9,10].

The inevitable mutual coupling between antenna elements in a MIMO system is important, which affects the antenna’s characteristics negatively. It disturbs the pattern correlation and radiation efficiency. The decoupling technique is a way to suppress the mutual coupling (enhance the isolation) in MIMO and array antennas. It has been utilized in various frequency bands and applications such as Long-Term Evolution (LTE) (3400–3800 MHz) and sub-6 GHz 5G (3400–3600 MHz) [11,12]. To meet the requirements of the communication system, a MIMO antenna system should have high stability while keeping the Half Power Beamwidth (HPBW) value low [13]. Several techniques exist to decrease the mutual coupling; some of them are through the application of neutralization lines [14,15], the addition of parasitic elements [16], and methods of decomposition [17,18,19]. Not all these techniques can be utilized in a MIMO system efficiently or may not be suitable for Massive MIMO systems. On the other hand, the use of metasurfaces and metamaterials in antennas has shown promising improvement by decreasing mutual coupling. However, they are limited by the number of elements that can be added to a design [20,21,22,23]. Some 5G antennas must be designed with compact dimensions as stated in [24,25,26,27,28,29,30]. Since single-mode antenna elements were used to form MIMO antennas in these designs, they offered limited gain, a low data rate, and a limited bandwidth (BW) [3].

This paper has four sections. First, a comprehensive introduction of the recent papers about MIMO antennas, their technologies, and the challenges related to the sub-6 GHz communications are presented in Section 1. Then, the proposed MIMO antenna and the metamaterial structure design procedures, and their performance investigation are shown in Section 2. When the antenna capabilities of working as a high gain MIMO antenna was assessed, four array elements of the proposed MIMO antenna were integrated on a single board to function as an eight ports MIMO antenna on a mobile handset. The results of this study are presented in Section 3. Finally, the concluding remarks are in Section 4.

## 2. Design and Characterization of the Proposed MIMO Antenna

The MIMO antenna should be designed carefully to overcome the mentioned problems. In this paper, the basic characteristics of a proposed design consisting of an antipodal-spiral antenna integrated with seven metamaterial elements are investigated and the antenna’s ability in working as a transceiver element for 5G communication applications is shown. First, an antipodal Vivaldi and a spiral antenna were designed, and their characteristics were assessed. These two antennas were integrated. Next, a metamaterial unit cell was designed, and its negative index properties at 5G applicable frequencies were extracted. The metamaterial elements were then added to the proposed antenna to improve the MIMO antenna characteristics and reduce the mutual coupling, therefore, improve the antenna’s gain and radiation efficiency.

### 2.1. Antipodal Antenna and Spiral Patch Antenna Design Configuration

**Figure 1:**
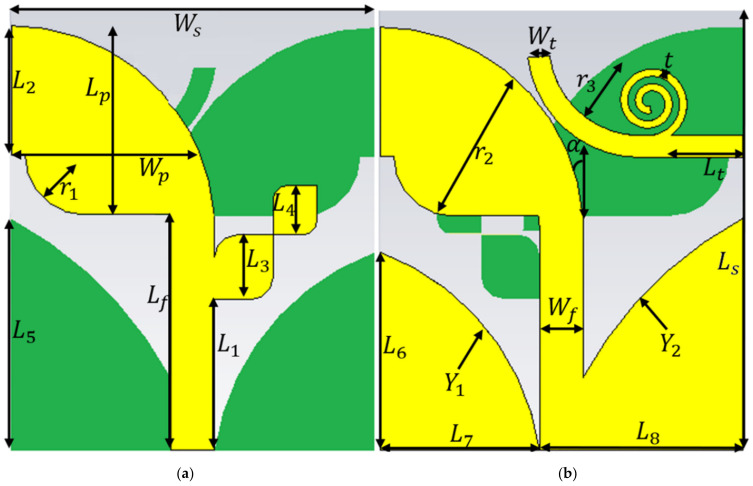
The simulated prototype of the proposed antenna: (**a**) front and (**b**) back view (both green and yellow colors are the conductors. Their color is different only to differentiate the front and back view of antenna).

The proposed antenna comprises a modified antipodal Vivaldi and a spiral patch element to achieve a circular polarization and enhanced directional pattern. The antenna structure and dimensions are presented in Figure 1 and Table 1. It is designed on a Rogers 5880 substrate with a dielectric constant of $\varepsilon_{r}=2.2$, loss tangent of tanδ = 0.0009, and thickness of h = 0.254 mm. Vivaldi antennas usually are resonance structures at low frequencies and work as traveling-wave radiators at high frequencies. The cut-off frequency at lower frequencies strappingly varies with the antenna’s width and it should be around λ/2 at the low-frequency band. Another factor that can influence the antenna’s performance is the substrate thickness. When it is too large, the undesired modes are excited accordingly. Increment and excitement of these unwanted modes distort pattern and increase in the cross-polarization level. Hence, a substrate with a small thickness and low dielectric constant is preferred. The phase difference of the traveling-wave currents can be defined by optimizing the electrical length of each radiating antipodal flare. Therefore, the electrical length of this flare influences the gain and determines the lower and higher cut-off frequencies of the antenna [31,32,33].

Antipodal Vivaldi Antennas (AVAs) have been proposed to improve the BW and have been used in numerous applications [34,35,36]. The transition between the feed line and the tapered slot should be defined carefully to avoid the reduction in the BW and gain as compared to the conventional Vivaldi antenna. Additionally, unlike conventional Vivaldi antennas, the AVAs offer low cross-polarization and radiation beam alteration [37]. Hence, the research effort is toward improving the performance of AVAs. For instance, in [38] authors proposed the use of a dielectric lens to focus the energy. The use of artificial materials was proposed in [39] and parasitic elliptical patch in [40]. In this paper, we propose a modified low-profile AVA design with robust gain and improved feeding method for 5G and X-band communications. The modifications consist of an exponential feed line and metamaterial (MTM) loadings. A full-wave electromagnetic simulation software, CST Microwave Studio (CST MWS) was employed to design the antenna and evaluate its performance. CST MWS uses the Finite Integration Technique (FIT).

Figure 1 depicts the model of the proposed AVA integrated with a planar spiral antenna. The AVA antenna is chosen due to its promising performance and high directional gain [41]. A conventional AVA was initially designed and simulated to operate at 3.8 GHz, then the initial dimensions were altered. The dimensions of the exponential curve and the tapered slot were computed using the equations presented in [42,43]. Two modified antipodal patches with exponentially tapered slots are forming the radiation area. The initial design parameters of the antenna are α angle, and the patch and feed line dimensions as $L_{p},\;L_{f},\;W_{f}$ and $W_{p}$, as shown in Figure 1. The actual dimensions of the exponential ground section were made following Equations (1) and (2) (x is the location of the points).

(1) $$Y_{1}=\;-\;\left(1.3^{x}+13.5\right)$$

(2) $$Y_{2}=\;-\;\left(0.67^{x}+15.8\right)$$

This shape is used for mutual coupling reduction, heterogeneous tapering, forming capacitive gaps for cross-polarization reduction, and to achieve a 50 Ω input impedance. Afterward, two cross-curved corner squares with sides of $L_{3}$ and $L_{4}$ were added to the opposite side of the feed line to create another resonance at 5.2 GHz.

The second antenna that was created on the same area and substrate is a planar spiral antenna fed through an exponential transmission line (a combination of a straight transmission line and a quarter circular ring with a radius of $r_{3}=\;6.25\;$ mm) with length and width of $L_{t}$ and $W_{t}$, respectively. Spiral antennas have circular polarization and can achieve wide BW while keeping the antenna dimensions small. Therefore, spiral antennas are good candidates for applications that require a wide BW [42]. The proposed spiral antenna was designed with the width of t = 0.25  mm, n = 1.5 turns, an internal radius of 0.5 mm, and a gap of 0.25 mm. The gap and width of the antenna should be designed carefully to avoid the increase in the surface wave levels. After optimizing both antennas to obtain the required resonance frequencies the two antennas were integrated to form one antenna with two operating modes (AVA: Ant 1) and (Spiral: Ant 2) capable of working as a dual-polarized triple-band antenna for 5G and X-band communication applications.

**Figure 2:**
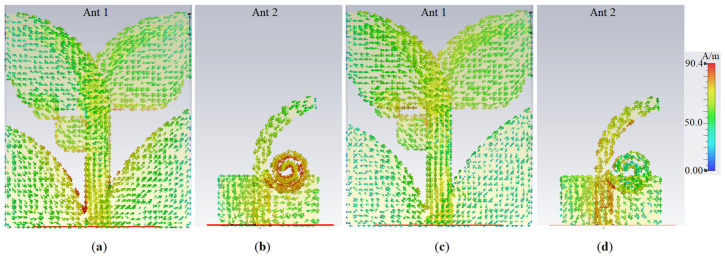
The surface current density for Ant 1 and Ant 2: (**a**) and (**b**) at 3.8 GHz; (**c**) and (**d**) at 5.2 GHz (red color is maximum and blue is the minimum).

Adding two rectangles at the junction of AVA and transmission line created one more resonance frequency, and reduced the coupling, but it shifted the resonance to a higher band. It also improved the impedance matching. The patches are rounded at the corner next to the substrate’s edges to suppress the fringing fields and enhance the antenna’s radiation efficiency. The effects of the spaces, the tapered angle, and the optimized dimensions were investigated by studying the surface current distribution at the desired frequencies. Figure 2 shows the surface current density (SCD) at desired operating bands (3.8 GHz, 5.2 GHz) for the proposed Ant 1 and Ant 2. For Ant 1, the surface current’s magnitude on the tapered ground next to the transmission line is the strongest, hence, two rectangles can act as an inductor to suppress the surface waves and coupling. On the other hand, Ant 2 shows the strongest current around the spiral patch at 3.8 GHz and around the exponential transmission line at 5.2 GHz. Furthermore, Figure 2 reveals that the SCD is sharper around the line of feeding, ground, and antipodal patches for Ant 1 and it shows more density around the spiral at 3.8 GHz for Ant 2.

**Figure 3:**
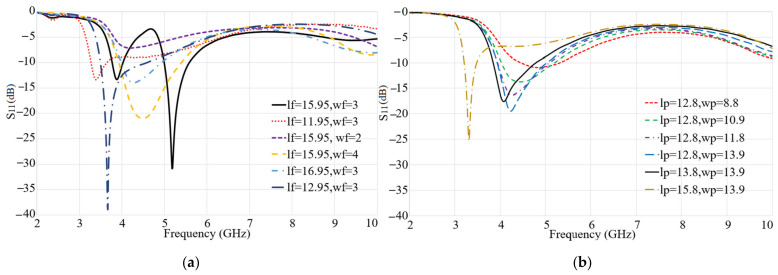
Parametric study of Antipodal Vivaldi Antennas (AVA) antenna: (**a**) lf ($L_{f}$): feed line length, wf ($W_{f}$): feed line width, (**b**) lp, and wp ($L_{p}$, $W_{p})$ are the patch’s length and width (dimensions are given in mm).

Figure 3a, b depict the variations in the reflection coefficient of the Ant 1 in terms of feed ($L_{f}$, $W_{f}$) and patch ($L_{p}$*,*$W_{p}$) dimensions, respectively. The results show that when the transmission line length is shorter than 15 mm ($L_{f}<15$ mm) the second band at sub-6 GHz is disturbed and vanished and the lower band is shifted to a lower frequency. The feed line’s width affects the impedance matching of the antenna. For instance, when $W_{f}<3$ mm and slightly more than 3 mm the resonance at the higher band is disturbed, and the lower band is shifted to a different frequency. The patch dimensions parametric sweeps are presented in Figure 3b. As depicted the increase in the patch length enhances the resonant frequency and moves it to a lower band. On the other hand, the patch width affects the bandwidth at the sub-6 GHz band as increasing the width decreases the resonance and its reduction reduces the working BW.

**Figure 4:**
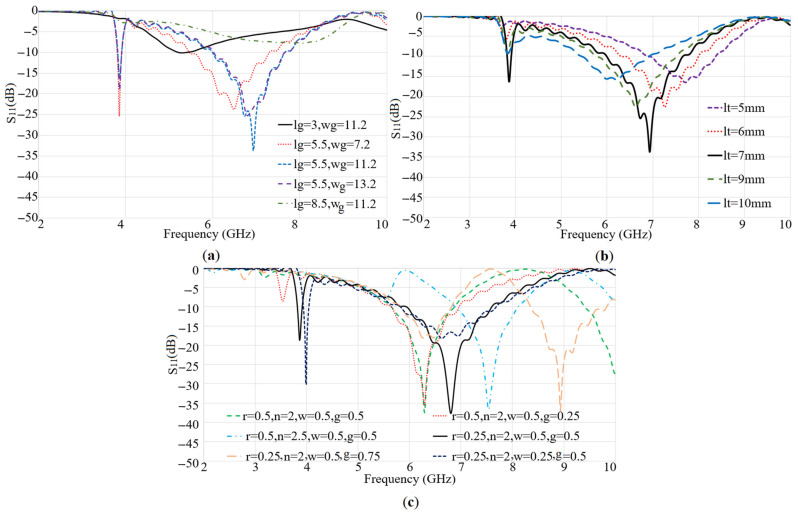
Parametric study of the spiral patch antenna dimensions: (**a**) lg: ground length (L_g_) and wg: width (W_g_), (**b**) lt: feed line length ($L_{t}$, (**c**) spiral dimensions: internal radius (r), number of turns (n), line width (w), and the gap between lines (g).

**Figure 5:**
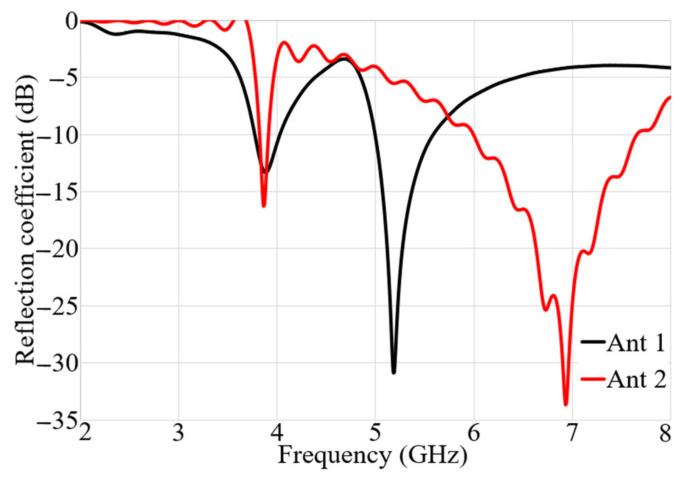
Ant 1 and Ant 2′s reflection coefficient results.

Figure 4 demonstrates the results of a parametric study of the important parameters of Ant 2 in terms of ground dimensions ($L_{g}$, W_g_) (Figure 4a), the exponential transmission line (first section only: $L_{t}$) (Figure 4b), and the spiral patch dimensions (Figure 4c) such as the internal radius (r), the turn (n), the line width (w), and the gap (g). Figure 4a indicates that the ground length and width of Ant 2 do not affect the resonance at the lower band (3.8 GHz). However, the increase in the ground length shifts the second resonance frequency to a lower value and when it exceeds 5.5 mm, the second resonance frequency is significantly reduced. The variation of the transmission line length ($L_{t}$) is presented in Figure 4b. This figure shows that the increase in this length does not alter the first resonance frequency much, but it shifts the higher resonance frequency to a lower value. An increase of the spiral’s internal radius disturbs the first resonance at 3.8 GHz and decreases the value of the second resonance frequency. Reducing the line’s width decreases the BW at the higher frequency band and shifts the first resonance frequency to a higher band. This might be because as the gap is reduced the surface waves around the spiral are reduced. The reflection coefficients of the resulting structure for Ant 1 and Ant 2 after optimizing the dimensions of both antennas are presented in Figure 5. The obtained resonance frequencies are at 3.8 GHz, 5.2 GHz for Ant 1, and 3.8 GHz and 6.9 GHz for Ant 2.

**Figure 6:**
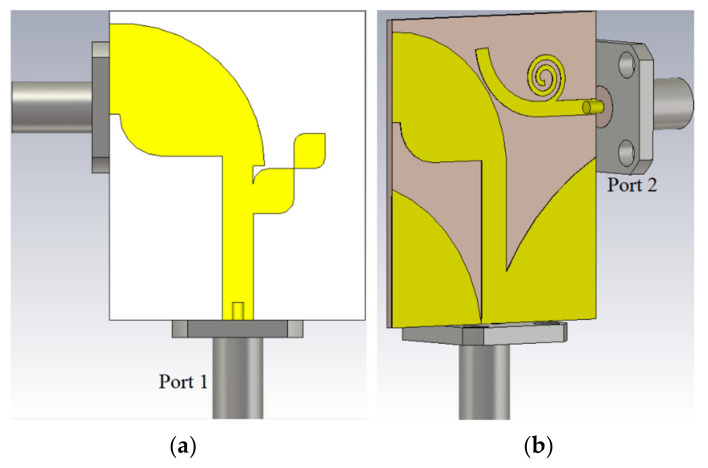
The proposed antenna’s (**a**) front, and (**b**) back prototype with SMA connectors.

Ant 1 and Ant 2 were integrated to create the proposed multiband dual-polarization antenna for resonance frequencies at 3.8 GHz and 5.8 GHz, as a part of a communication system for 5G X-band applications. Figure 6 shows the 2D and 3D views of the proposed antenna after integration with (SubMiniature version A) SMA connectors.

**Figure 7:**
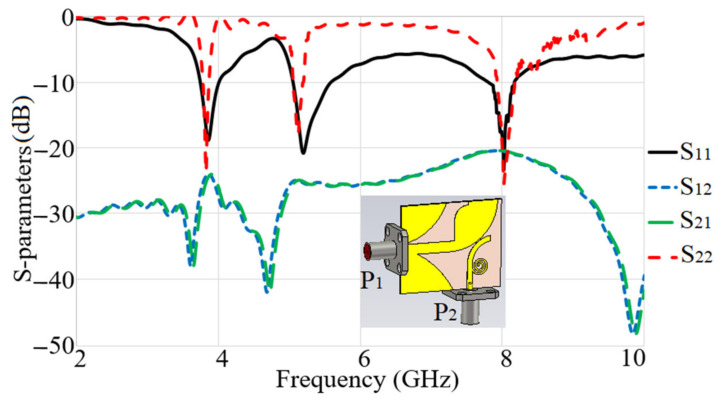
Simulation results of S-parameters for the proposed dual-polarized antenna (P1 and P2 are port 1 and port 2).

The two modes of operation, Ant 1 and Ant 2, provide vertical and horizontal polarizations, respectively. To match the feed lines’ width $W_{f}$ and $W_{t}$ are considered. $L_{f}$ and $L_{t}$ also affect the matching. To consider the effect of the connectors, we added the SMA connectors in the simulations, as shown in Figure 6. To attain sufficient distance between the two SMA connectors, the feed lines’ length was increased to 5 mm in both x and y directions. Since two radiation modes (Ant 1 and Ant 2) are orthogonally polarized high isolation is anticipated. The simulated reflection and the transmission coefficients of the proposed antennas are shown in Figure 7. The low values of the transmission coefficients show a high-level of isolation between the two ports within the operating bandwidth (S_21_ less than −20 dB) and the antenna has a good matching (S_11_ and S_22_ less than −10dB). The antenna shows matching at three frequencies for the two modes of operation Ant 1 and Ant 2, which are 3.8 GHz with a BW of almost 300 MHz, 5.2 GHz with a BW of 550 MHz, and 8.0 GHz with a BW of 300 MHz.

**Figure 8:**
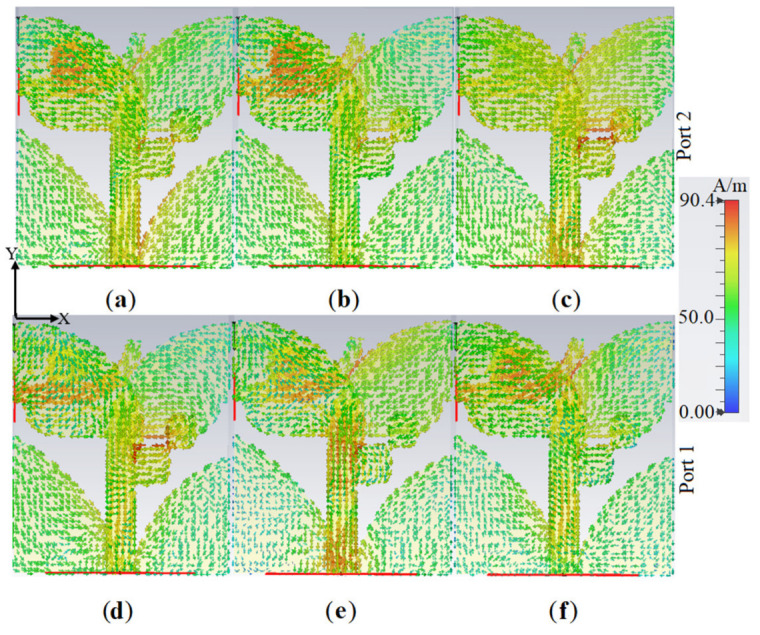
The proposed antenna’s surface current density (SCD) variations: (**a**,**d**) 3.8 GHz, (**b**,**e**) 5.2 GHz, and (**c**,**f**) 8.0 GHz) (red color is maximum and blue is the minimum).

**Figure 9:**
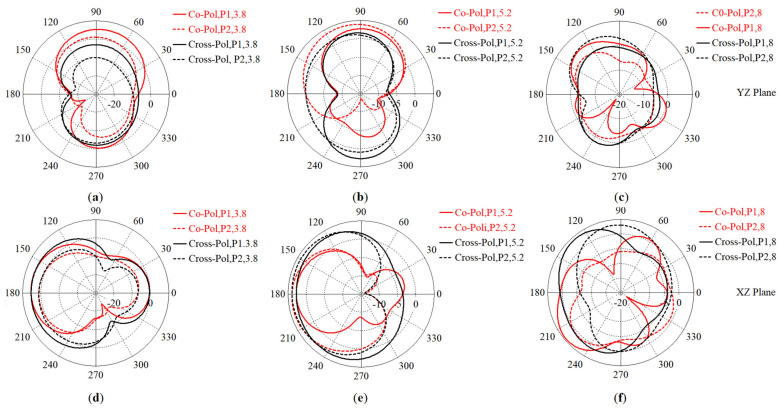
The simulated 2D co-polarization and cross-polarization radiation patterns of the proposed antenna without on the yz and xz planes: (**a**) and (**d**) 3.8 GHz, (**b**) and (**e**) 5.2 GHz, and (**c**) and (**f**) 8.0 GHz.

**Figure 10:**
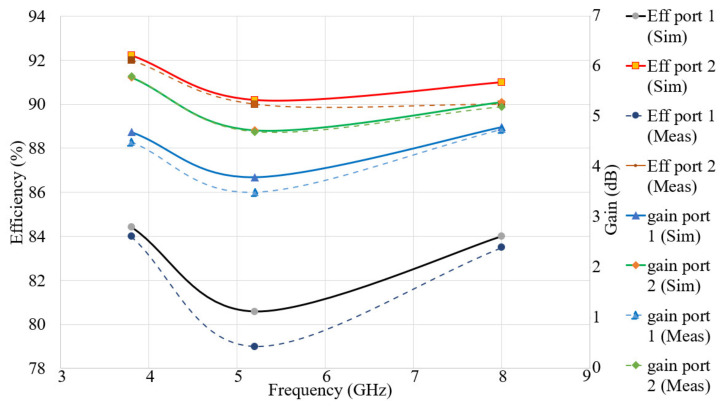
The radiation efficiency and simulation and measurement gain of the proposed Multiple Input Multiple Output (MIMO) (Gain in dBi, Eff: Efficiency in%).

Figure 8 shows the SCD of the two modes (activating port 1 and port 2 for modes of Ant 1 and Ant 2, respectively) at the three resonance frequencies. The proposed antenna’s SCD at 3.8 GHz, 5.2 GHz, and 8.0 GHz for the two ports were studied to ensure the achievement of dual-polarization operation. Figure 8 shows that the SCD flows along y-axis when port 1 is active, while the current flows towards x-axis when port 2 is active. The 3D radiation patterns of the proposed antenna for Ant 1 and Ant 2 modes for the three working frequencies are presented in Figure 9. It can be observed that the end-fire 3D radiation pattern is not altered drastically for Ant 1 and Ant 2 at the resonance frequencies. The antenna attains a maximum gain and radiation efficiency of 5.39 dBi and 95% at 3.8 GHz, respectively. Figure 10 and Table 2 display the radiation efficiency and gain of the proposed antenna for Ant 1 and Ant 2 at 3.8 GHz, 5.2 GHz, and 8.0 GHz.

### 2.2. Single Element Metamaterial Antenna and the Array Configurations

Metamaterials (MTM) are made using periodic structures. They are applied to acquire wide bandwidth and higher directive gain while maintaining a low-profile structure. We consider using MTM in a planar format in the direction of end-fire radiation.

**Figure 11:**
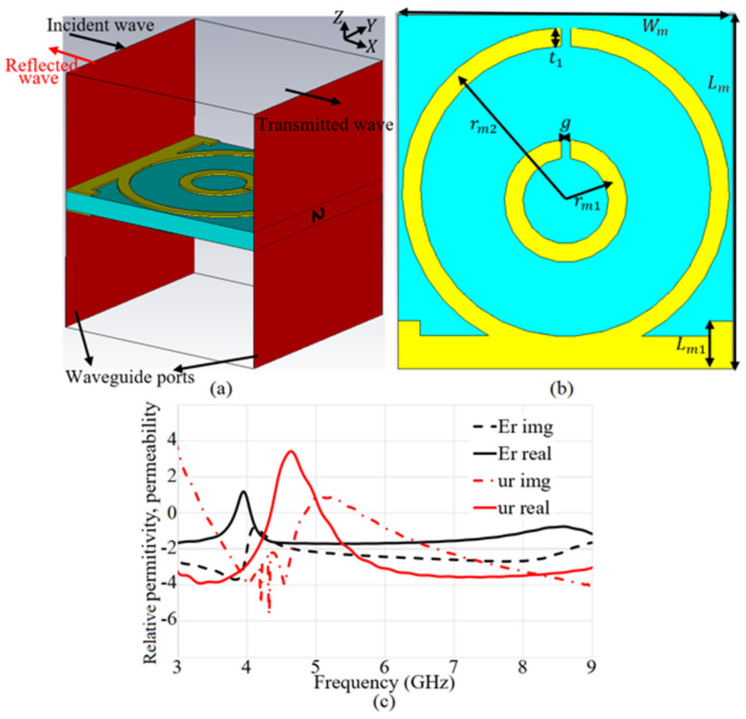
Metamaterials (MTM) structure’s parameters and permittivity/permeability results: (**a**) 3D view of MTM structure, (**b**) 2D view, (**c**) relative permittivity (E_r_) and permeability (u_r_).

Figure 11a shows the proposed MTM unit cell. A Split-Ring Resonator (SRR) structure containing two concentric circular rings is considered for the preliminary design of the MTM unit cell [43]. The SRR is assumed as a magnetical resonator that is influenced by the vertical magnetic field to produce permeability [44,45]. Conversely, the splits (gaps) are normally cut out of the structure to create a capacitance and to monitor the structure’s resonant specification. Adding a Capacitive-Loaded Strip (CLS) is used to tune the MTM structure to operate in the required frequency band. It should be mentioned that a CLS works similar to an electric dipole [46]. The arrangement of the rings and strips together presents electric (E) and magnetic (H) fields and the CLSs create additional parallel E-fields. The proposed MTM structure is placed on both sides of the substrate.

The MTM unit cell shown in Figure 11a, b is designed on a polytetrafluoroethylene (PTFE) substrate with a $\varepsilon_{r}=2.55$, tanδ = 0.001, and a thickness of h = 0.6 mm. The MTM structure is surrounded by perfect magnetic and perfect electric boundaries (xz and yz planes, respectively). Two waveguide ports are added to the two faces of the cell parallel to the xy planes. An open boundary is assumed towards the z-axis. The Nicolson–Ross–Weir (NRW) method, reported in [47], was performed to examine the electromagnetic specifications of the proposed MTM unit cell to determine and then export the relative permittivity ($\varepsilon_{r}$), relative permeability ($\mu_{r}$), and the refractive index ($n_{r}$) using the simulated reflection and transmission coefficient (Figure 11c). The operating BW range of $\varepsilon_{r}$, and $\mu_{r}$ are summarized in Figure 11. Afterward, seven units of the proposed MTM were incorporated with the antenna to improve the antenna’s performance such as gain, directivity, radiation efficiency, and impedance matching through the suppression of surface waves occurred due to the reduction of the tapered angle between two patches of MAVA. The MTM elements were rotated in different directions with steps of 90°, as shown in Figure 11.

After the optimal dimensions of the modified antipodal Vivaldi antenna were obtained, to improve the antenna’s performance while keeping a small footprint, the angle (α) was reduced to create more concentration towards the end-fire direction and decrease the antenna’s width. However, this reduction increased the surface waves, which also affected the antenna’s performance.

### 2.3. Design and Characterization of MIMO Antenna Integrated with MTM Structure

**Figure 12:**
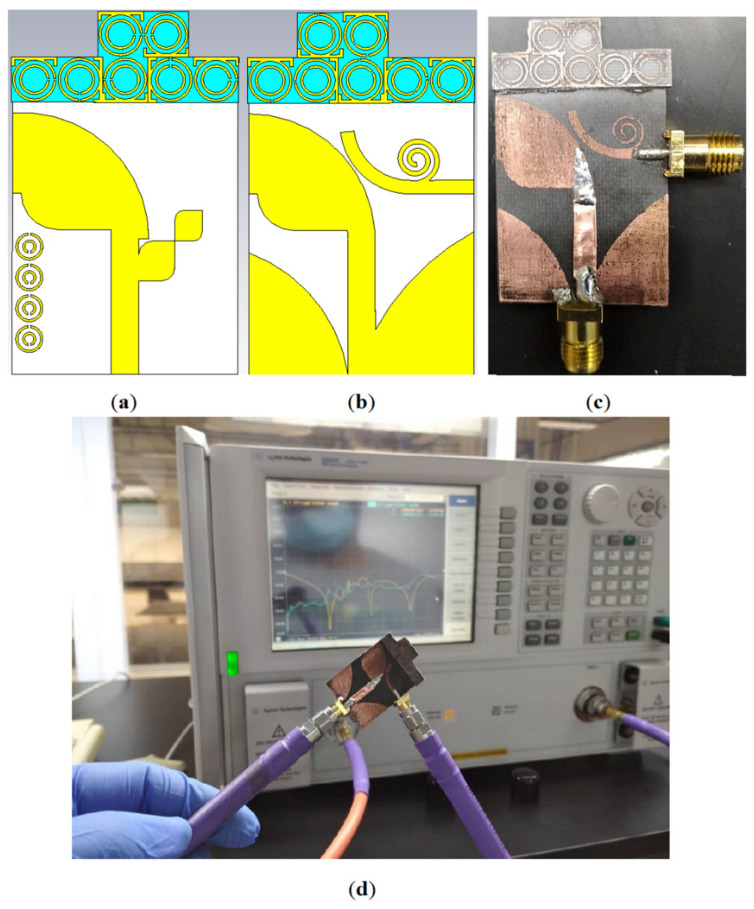
The AVA antenna integrated with the MTM elements: (**a**,**b**) the simulated structure, (**c**) the measured prototype, and (**d**) the measurement setup.

In this section, we present the results of the integration of the proposed MIMO antenna with seven elements of the modified SRR MTM, after reducing the antenna width (to miniaturize the antenna). Table 3 and Table 4, and Figure 11 show the optimized dimensions of the antenna after integration with MTM. Figure 12 depicts the simulated and fabricated prototypes of the proposed antenna integrated with MTM elements. After adding the MTM elements, the total size of the antenna was increased by 10 mm in y direction.

**Figure 13:**
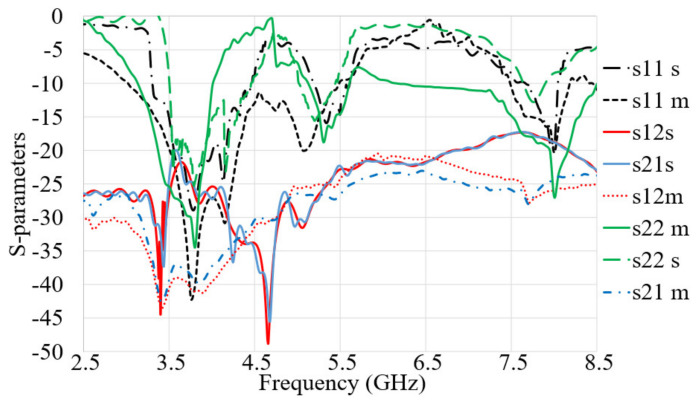
The simulated and measured S_11_, S_12_, S_21_, and S_22_ results of the proposed antenna (s: Simulation, m: Measurement).

Figure 13 shows the simulated and measured S-parameters (reflection and transmission coefficients). The measurement was performed using a network analyzer, PNA model HP 85070-Ds. The PNA was calibrated for the frequency range of 100 MHz–10 GHz. Figure 13 does not show any significant difference between the simulated and measured S-parameters. These results verified that the proposed antenna accomplishes broad BW that meet the BW required for the 5G and X-band communication. In addition to that, acceptable isolation exists between the two ports (S_21_ less than −20 dB).

**Figure 14:**
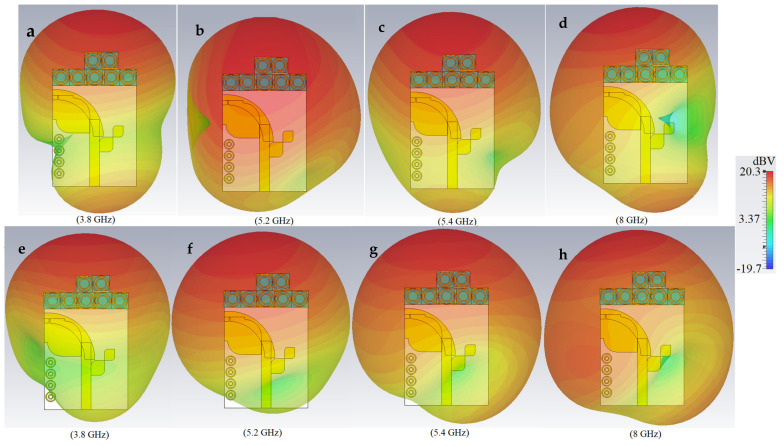
The 3D E-pattern of the proposed antenna integrated with MTM elements (**a**), and (**e**) at 3.8 GHz; (**b**) and (**f**) at 5.2 GHz; (**c**) and (**g**) at 5.4 GHz; and (**d**) and (**h**) at 8.0 GHz (red color is maximum and blue is the minimum).

**Figure 15:**
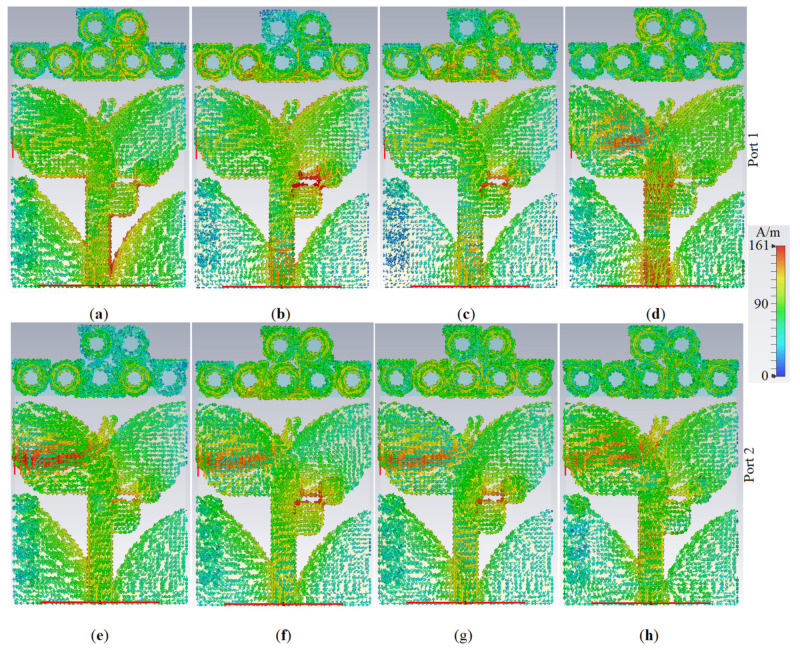
The SCD variation of the proposed antenna: (**a**,**e**) at 3.8 GHz; (**b**,**f**) at 5.2 GHz; (**c**,**g**) at 5.4 GHz; (**d**,**h**) at 8.0 GHz (red color is maximum and blue is the minimum).

Figure 14 and Figure 15 depict the simulated 3D radiation patterns and SCD of the proposed antenna after MTM addition, respectively, when either Ant 1 (port 1) or Ant 2 (port 2) are active, at frequencies of 3.8 GHz, 5.2 GHz, 5.4 GHz, and 8.0 GHz.

**Figure 16:**
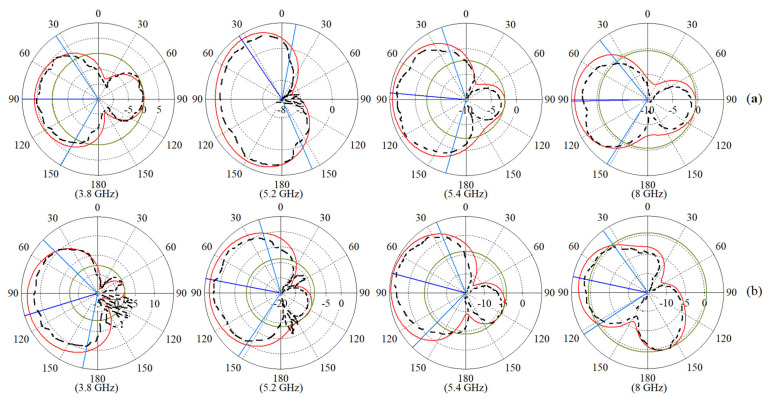
The 2D radiation patterns of the antenna for: (**a**) Ant 1, (**b**) Ant 2 (red line: simulated co-polarization, black dashed line: measured co-polarization, on the xz plane).

The simulated and measured radiation patterns of the antenna (co-polarization on the xz plane) are depicted for Ant 1 and Ant 2 at the resonance frequencies (Figure 16). The cross-polarization discrimination is better than 20 dB. Furthermore, the proposed MTM-integrated antenna achieved lower back radiation and side lobes for each mode of radiation than those for the original antenna.

## 3. Proposed MIMO Antenna for Smartphones

**Figure 17:**
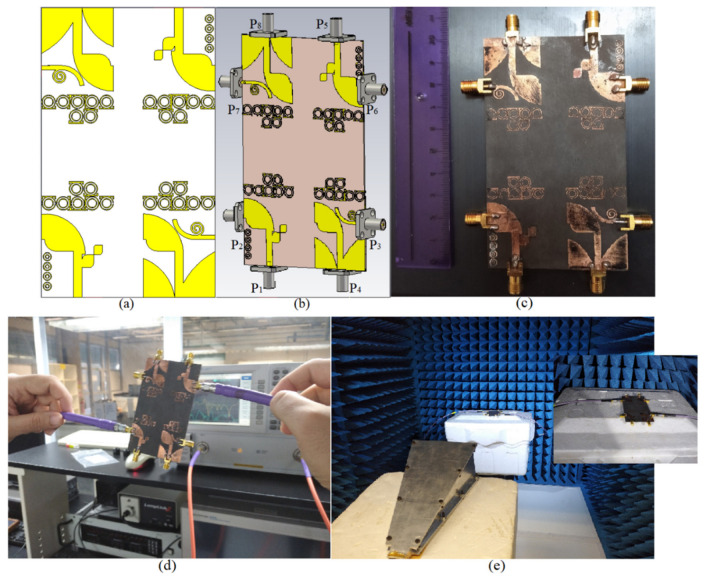
The MIMO antennas on a board with the same size as a smartphone: (**a**) 2D view of the prototype of the MIMO antenna, (**b**) 3D view of the structure with the SMA connectors, (**c**) fabricated MIMO for smartphone, (**d**) S-parameter measurement setup, and (**e**) radiation pattern measurement setup.

**Figure 18:**
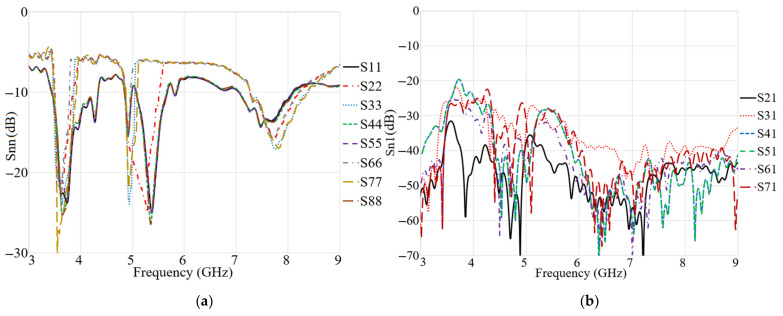
The simulated: (**a**) reflection and (**b**) transmission coefficient results of the MIMO antenna for a handset structure.

To create a 2 × 2 MIMO arrangement utilizing the proposed antenna the antenna elements were placed at the four corners of a board similar to the smartphone board with an overall dimension of $60\;\times \;100\;{\mathrm{mm}}^{2}$ (width × length), as presented in Figure 17a,b. The fabricated handset MIMO antenna and its measurement set up in the air are shown in Figure 17c,d. To measure the S-parameter of the handset, port 1 is connected to terminal one of the PNA and the other ports are connecting to terminal 2 of the PNA (P_1_ is the transmitter and P_2_–P_8_ are the receivers). The radiation pattern measurement setup of the handset MIMO antenna is presented in Figure 17e. Figure 18a depicts the simulated reflection coefficients for the MIMO antenna with eight ports. The transmission coefficients related to Ant 1 (ports 1, 3, 5, 7) of the antennas are plotted in Figure 18b. The transmission coefficient results show a level of less than −20 dB even for the worst case that was between port 1 and port 5. The handset antenna can achieve maximum operating BW from two ports (1.35 GHz for port 1 and 1 GHz for port 2) and maximum shared BW of more than 1 GHz among all ports. The proposed MTM-based antenna’s radiation efficiency is around 89% within the entire frequency band. It can be noticed that the MTM array elements are used to enhance the proposed antenna in order to accomplish a maximum gain and efficiency of 13.5 dBi and 98%, respectively. The results from the other ports are approximately similar to a single antenna with two modes of Ant 1 and Ant 2.

**Figure 19:**
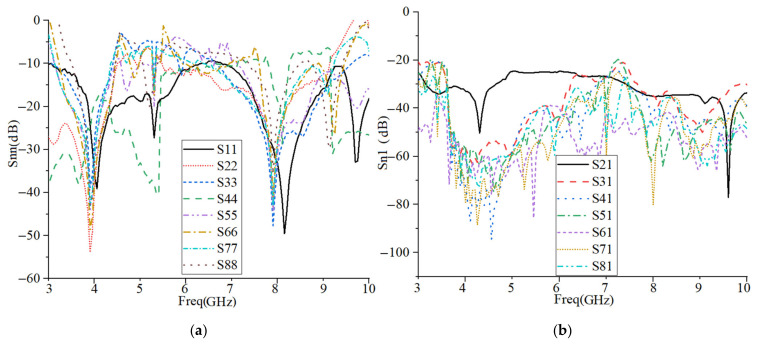
Measured: (**a**) reflection and (**b**) transmission coefficients of the MIMO antenna.

Figure 19 indicates the experimental results for reflection and transmission coefficients attained by the handset MIMO using a board with a similar size to a smartphone. It depicts that the MIMO antenna works well at the frequencies of 3.8 GHz, 5.2–5.4 GHz, and 5.8 GHz. Besides, good isolation between the ports exists as the transmission coefficients of port 1 and ports 2–8 are shown. The measured transmission coefficient follows the same trend as the simulation result.

Envelope Correlation Coefficient (ECC) is one of the key factors for the performance evaluation of a MIMO antenna system. It is used to determine how similar the antenna elements perform in a MIMO system and to define the diversity among them. ECC can be acceptable when it has a value of less than 0.5 [48,49,50]. When ECC is small, high isolation between every pair of antenna elements exists. ECC can be defined using the radiation pattern of the antenna as shown in Equation (3):

(3) ρmn=|∫∫04π[Fm→θ,∅×Fn→θ,∅]dΩ|2∫∫04πFm→θ,∅2dΩ∫∫04πFn→θ,∅2dΩ

where $\rho_{mn}$ is the value of ECC between the m and n ports, $\vec{F_{m}}\left(\theta ,\emptyset \right)$ is the radiation patterns of the antenna. In our case n and $m\epsilon \left\{1,2,\cdots ,8\right\}$.

**Figure 20:**
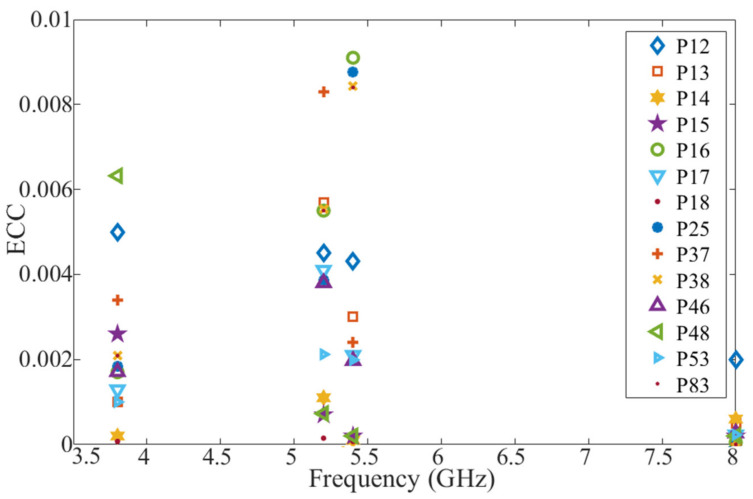
Envelope Correlation Coefficient (ECC) variation between different pairs of ports (e.g., P12: ECC between Port 1 and Port 2).

ECC values of the MIMO antenna elements utilizing the 3D radiation of each element are depicted in Figure 20. It should be mentioned that for each case a pair of MIMO elements are considered. Very small ECC values are observed, as expected, due to the orthogonality of the polarization between every two adjacent antennas. The ECC values less than 0.01 prove that the MIMO antennas in the handset provide good diversity performance. (The presented ports in Figure 20 have been chosen as examples).

### Specific Absorption Rate

The Specific Absorption Rate (SAR) is another key factor that shows how much power is absorbed by the human tissues and it should be assessed when a MIMO antenna is used for a smartphone [51,52,53]. The SAR is defined as the absorbed energy by a unit mass of tissue. Two major standards are the American and European ones. The first one restricts the SAR to 1.6 W/kg for every 1 g of tissue, and the latter requires the SAR value to be less than 2 W/kg for every 10 g of tissue [54].

**Figure 21:**
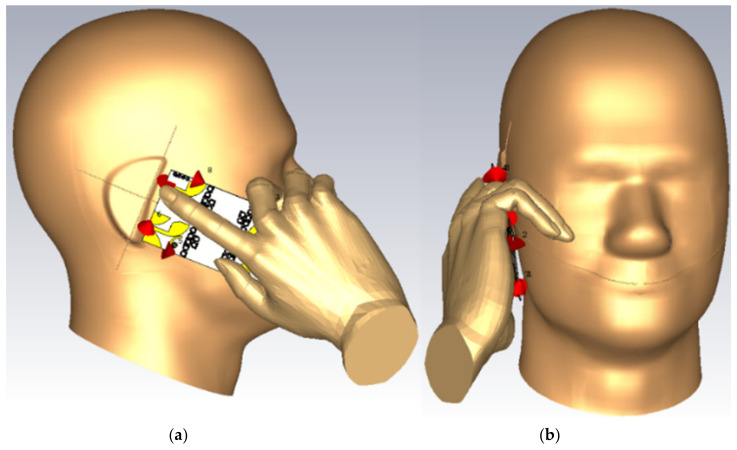
The proposed handset MIMO on the human head model: (**a**) side view, and (**b**) front view.

**Figure 22:**
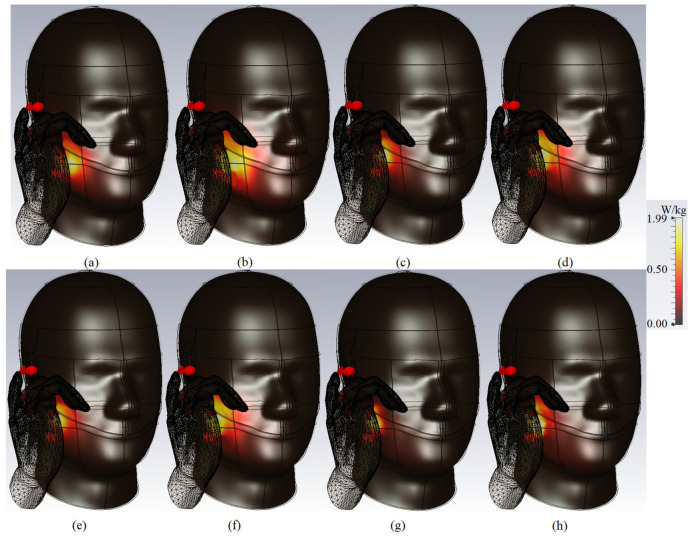
Specific Absorption Rate (SAR) distribution for MIMO elements on head and hand models: (**a**) 1g and (**b**) 10 g at 3.8 GHz; (**c**) 1g and (**d**) 10g at 5.2 GHz; (**e**) 1g and (**f**) 10 g at 5.4 GHz; (**g**) 1g and (**h**) 10g at 8.0 GHz.

Figure 21 and Figure 22 show the simulation setup of the handset with MIMO antenna for simulation and calculation of the distribution of SAR due to an 8 element MIMO antenna. Calculations were done for 1 g and 10 g tissues at 3.8 GHz, 5.2 GHz, 5.4 GHz, and 8 GHz, respectively. The SAM phantom head and hand model in CST MWS was used. The head model consists of the shell and the fluid. The shell’s average tissue with the properties are $\varepsilon_{r}$= 3.7, $\mu_{r}=\;1,\;\sigma =0.0016$ S/m, ρ=1000 kg/m^3^ where σ is the tissue conductivity and ρ is the tissue mass density. The fluid has a dispersive permittivity and $\mu_{r}=\;1,\;\rho =1000$ kg/m^3^. The hand model has an average tissue material with the properties of $\varepsilon_{r}$= 20.0, $\mu_{r}=\;1,\;\sigma =1$ S/m, ρ =1000 kg/m^3^.

The detailed SAR values are depicted for both standards in Table 5. The handset MIMO is considered in the vicinity of the human head and hand models. It should be mentioned that the input power of the proposed antenna elements at operating frequencies is assumed to 24 dBm for every element.

The simulated SAR shown in Figure 22 indicate acceptable SAR values at the desired frequencies. Figure 22a,c,e,g are the SAR values for 1g standard at 3.8 GHz, 5.2 GHz, 5.4 GHz, 8 GHz, respectively. Figure 22b,d,f,h are the SAR values for the 10 g standards at 3.8 GHz, 5.2 GHz, 5.4 GHz, and 8 GHz, respectively. The antenna has the proximity of 1 mm space and an inclined angle of 65°.

Table 6 and Table 7 are the comparisons between the proposed MIMO antenna and a few other reference papers. They show that the proposed MIMO antenna and its integration in smartphones provide better performance in terms of isolation, gain, operating frequency bandwidth, radiation efficiency, and the ECC. The proposed design offers high isolation among its ports, high gain and efficiency, and a very thin structure in comparison with all the antennas from the references listed in Table 6 and Table 7. The proposed work presents a comprehensive study of the MIMO antennas for smartphone applications.

**Figure 23:**
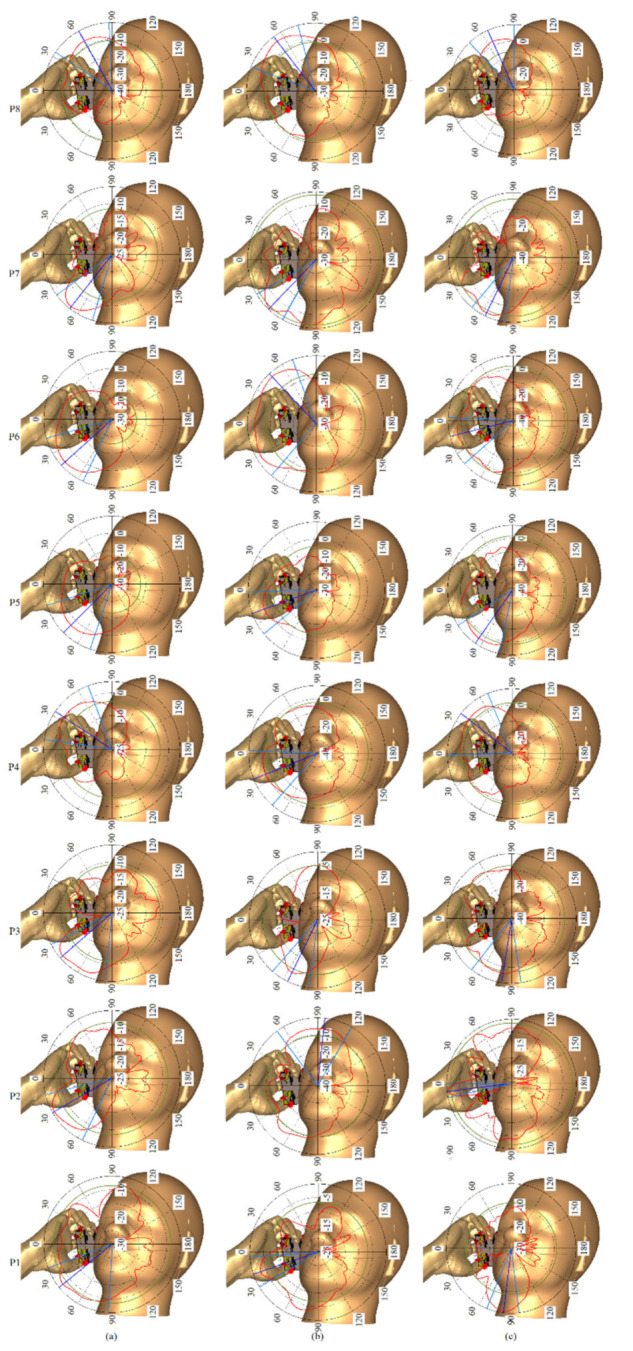
The 2D radiation pattern of the proposed MIMO with head and hand model: (**a**) at 3.8 GHz, (**b**) 5.2 GHz, and (**c**) 8.0 GHz.

**Figure 24:**
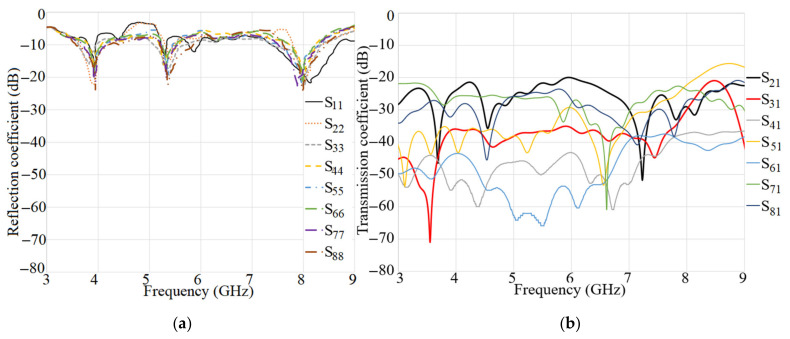
S-parameters of proposed MIMO antenna with head and hand models: (**a**) reflection coefficient and (**b**) transmission coefficient.

Further simulations were performed to examine the performance of the proposed MIMO antenna when it was held close to the human head using the head and hand model in CST MWS. Figure 23 shows the antenna patterns at each port and three resonance frequencies of 3.8 GHz, 5.2 GHz, and 8.0 GHz. It shows that the head and hand model affected the vertical polarization patterns more than the horizontal polarization patterns since the phone encountered the head and hand by its length mostly. Figure 24 shows that more than 20 dB isolation is obtained in the presence of the head. Besides, all three bands exist, but there is a slight frequency shift.

## 4. Conclusions

An MTM-based triple-band MIMO antenna was designed and investigated for utilization in 5G and X-band communications. Eight antenna elements were placed at the four corners of a smartphone board to be used for the communication links. The proposed metamaterial-integrated MIMO antenna comprises a modified antipodal Vivaldi antenna (Ant 1) and a planar spiral (Ant 2) to create dual-polarization and polarization diversity. The antenna was designed and mounted on a Rogers 5880 substrate with $\varepsilon_{r}$ = 2.2, tanδ= 0.0009, and the thickness of h = 0.254 mm. The dual-polarization capability of the proposed antenna is to reduce the high attenuation that occurs in the 5G communication systems and to obtain high data rates. In addition, the orthogonal polarization between every two antenna ports is utilized to attain high isolation among antenna ports. The proposed MIMO antenna offers an impedance matching bandwidth at 3.8 GHz, 5.2 GHz, and 8.0 GHz. The antenna is combined with the MTM structure to improve the antennas’ gain, and efficiency by suppressing the mutual coupling effects. A layered human head and hand model was considered in the simulation and the SAR values at all three resonances were examined.

In summary, high isolation, low profile, low complexity, compact size, high efficiency, high gain, and low cross-polarization were achieved by the proposed MIMO antenna and proved that it can be a good candidate for 5G, LTE, and X-band communications. Manufacturing of 5G devices and antennas using printed circuit board (PCB) technology faces many challenges, especially fabrication of high frequency and mm-wave bands antennas and integration of them with the radio frequency circuits. We proposed a thin and compact MIMO antenna that is suitable for ultra-thin 5G mobile devices. In addition to the compact size, the feeding and grounding of the antenna provide easy integration with electronic elements such as Micro-Electro-Mechanical Systems (MEMs). The integration provides means of designing the proposed antenna in a reconfigurable format to provide additional functionalities, which will be the future direction of this work.

## Figures and Tables

**Table 1:** The proposed antenna’s optimized dimensions.

Parameters	Dimensions (mm)	Parameters	Dimensions (mm)
$L_{s}$	30	$L_{8}$	13.9
$L_{f}$	15.95	$L_{p}$	12.8
$L_{t}$	7	$W_{p}$	13.9
$L_{1}$	10.3	$W_{s}$	24.8
$L_{2}$	8.85	$W_{f}$	3
$L_{3}$	4.4	$W_{t}$	1.5
$L_{4}$	3.4	$r_{1}$	4.25
$L_{5}$	15.8	$r_{2}$	12
$L_{6}$	13.5	$r_{3}$	6.25
$L_{7}$	10.9	t	0.5

**Table 2:** The radiation efficiency and gain of the proposed MIMO antenna at the resonance frequencies (Sim: Simulation, Meas: Measurement).

$f_{r}\;(\mathrm{GHz})$	Gain (dBi) Port 1 Sim/Meas	Gain (dBi) Port 2 Sim/Meas	Eff (%) Port 1 Sim/Meas	Eff (%) Port 2 Sim/Meas
3.8	4.70/4.50	5.78/5.80	84.42/84.00	92.00/91.00
5.2	3.80/3.50	4.73/4.70	80.58/79.00	90.19/89.50
8.0	4.80/4.75	5.29/5.20	84.00/83.50	91.00/90.00

**Table 3:** MTM structure dimensions.

Parameters	Dimensions (mm)
$L_{m}$	5
$L_{m1}$	1
$W_{m}$	5
$r_{m1}$	1.4
$r_{m2}$	2.2
$t_{1}$	0.35
g	0.25

**Table 4:** The MTM structure’s characteristics.

Parameters	$\mathrm{Relative}\;\mathrm{Permittivity}\;(\varepsilon_{r})$	$\mathrm{Relative}\;\mathrm{Permeability}\;(\mu_{r})$
Operating BW (GHz)	0–3.8, 4.1–9.0	0–4.2, 5.35–9.0

**Table 5:** Maximum SAR values (W/kg) at each frequency.

Port 1/*f_r_*	3.8 GHz	5.2 GHz	5.4 GHz	8 GHz
SAR 1g	0.526	0.723	0.990	1.091
SAR 10 g	0.391	1.121	1.540	1.991

**Table 6:** Comparison between a few reference antennas and the proposed antenna for 5G communication.

Reference Number	Dimensions (mm)	Max Isolation (dB)	Max Gain (dBi)	Band (GHz)	Efficiency (%)	Max ECC
[8]	75 × 25	20	5.3	5.29–6.12, 26.0–29.5	73	0.05
[55]	58 × 40	15	6.59	4.20–5.25	-	-
[56]	74 × 74	14.9	9.41	3.45–3.55	64	0.1192
[57]	150 × 150	35	8.5	2.40–4.26	-	-
[58]	45.8 × 52.9	<20	-	3.3–4.2	88	<0.1
[59]	50 × 100	25	>3	2.7–3.6	92	<0.009
Proposed	24.8 × 40	>22	6.75	3.30–4.45, 4.7–5.3, 7.7–8.25	91	<0.089

**Table 7:** Comparison of the performance of a few MIMO antennas for 5G smartphones.

Reference Number	Phone Board (mm^2^)	MIMO Order	Max Gain (dBi)	Max Isolation (dB)	Efficiency (%)	Max ECC
[4]	100 × 60	8	11	40	90	0.001
[6]	136 × 68	4	7.2	<60	-	-
[60]	75 × 150	8	4	25	>70	-
[61]	150 × 75	8	4	11.5	85	<0.08
[62]	68 × 136	8	-	>18	~60	<0.15
[63]	150 × 80	10	-	>11	<82	<0.2
Proposed	100 × 60	8	13.5	<40	94	0.009
